# Direct-to-Consumer Promotion of Prescription Drugs on Mobile Devices: Content Analysis

**DOI:** 10.2196/jmir.7306

**Published:** 2017-07-04

**Authors:** Kathryn J Aikin, Helen W Sullivan, Suzanne Dolina, Molly Lynch, Linda B Squiers

**Affiliations:** ^1^ Office of Prescription Drug Promotion Center for Drug Evaluation US Food and Drug Administration Silver Spring, MD United States; ^2^ RTI International Research Triangle Park, NC United States

**Keywords:** direct-to-consumer promotion, direct-to-consumer advertising, mobile devices, prescription drugs

## Abstract

**Background:**

US Food and Drug Administration (FDA) regulations state that any prescription drug promotion that presents drug benefits to consumers must also disclose certain information about the drug’s risks in a similar manner. Nearly three-quarters of all US mobile phone subscribers use a smartphone, and over half report receiving mobile advertisements on their device.

**Objective:**

The objective of this project was to investigate how prescription drugs are being promoted to consumers using mobile technologies. We were particularly interested in the presentation of drug benefits and risks, with regard to presence, placement, and prominence.

**Methods:**

We analyzed a sample of 51 mobile promotional communications and their associated linked landing pages. We assessed the content and format of the mobile communications and landing pages with regard to presentation of drug benefits and risks.

**Results:**

Of the 51 mobile communications we coded, 41% (21/51) were product claim communications (includes the drug name, benefits, and risks), 22% (11/51) were reminder communications (includes drug name only), and 37% (19/51) were help-seeking communications (includes information about the medical condition but not the drug name). Some of the product claim communications (5/21, 24%) required scrolling to see all the benefit information; in contrast, 95% (20/21) required scrolling to see all the risk information. Of the 19 product claim communications that presented both benefits and risks, 95% (18/19) presented benefits before risks and 47% (9/19) used a bigger font for benefits than for risks. Most mobile communications (35/51, 69%) linked to branded drug websites with both benefits and risks, 25% (13/51) linked to a landing page with benefits but no visible risks, and 6% (3/51) linked to a landing page with risks but no visible benefits. Few landing pages (4/51, 8%) required scrolling to see all the benefit information; in contrast, 51% (26/51) required scrolling to see all the risk information. Of the 35 landing pages with both benefit and risk information, 71% (25/35) presented benefits before risks and 51% (18/35) used a bigger font for benefits than for risks.

**Conclusions:**

These results indicate that, while risks and benefits are both represented in mobile communications and their associated landing pages, they are not equally prominent and accessible. This has implications for compliance with FDA fair balance regulations.

## Introduction

Information, entertainment, and advertisement consumption has been steadily moving away from print and network television stations to more specialized and targeted digital outlets, including Internet and mobile devices. By 2012, half of all mobile subscribers in the United States owned a smartphone [1], jumping to nearly three-quarters within 3 years [2]. In 2013, 57% of US smartphone owners reported receiving mobile ads at least once a day [3]. Prescription drug direct-to-consumer (DTC) promotion expenditure on the Internet experienced triple-digit growth between 2005 and 2009, compared with a decrease in other media channels [4]. One report [5] estimated that 3 billion to 7 billion pharmaceutical and drug promotional messages were being delivered online each month. Another noted that US health care and pharmaceutical advertisers spent US $373 million on mobile formats in 2014 [6]. Nevertheless, prescription drug promotion, while well represented on the Internet in the form of websites, has been slower to branch into mobile promotion beyond paid searches. One possible cause of this could be the issuance of US Food and Drug Administration (FDA) “enforcement” warning and untitled letters about sponsored links on Internet search engines in 2009 [7], which stated that online promotion is subject to existing laws and implementing regulations [8,9]. In particular, the US Food, Drug, and Cosmetic Act and implementing regulations state that any prescription drug promotion that presents drug benefits to consumers must also disclose certain information about the drug’s risks in a similar manner. This concept of “fair balance” may be challenging in mobile promotional communications (hereafter referred to as “mobile communications”), where there are often limitations on the amount of information that can be presented.

Content analyses have found fair balance lacking in DTC marketing in print [10-12], television [11,13-15], websites [16-18], social media sites [19], and Internet banners [20]. It is also important to have an accurate picture of the mobile promotion landscape in order to make informed decisions about mobile DTC promotion. The purpose of this content analysis was to explore the presentation of drug benefits and risks in (1) mobile communications and (2) landing pages (ie, the first page of the website to which the communication linked).

## Methods

After evaluating the capabilities of several companies, we chose to use a commercially available dataset of mobile-enhanced DTC promotional communications from Competitrack (Market Track, LLC, Chicago, IL, USA). Competitrack monitors 100 leading apps and mobile-optimized websites, including mobile devices—specifically, iPhones, Android phones, iPads, and Android tablets—by sampling hundreds of top-rated apps and websites to identify those that carry the most distinctive and requested consumer advertising. This media universe is reviewed on a regular basis to identify emerging apps and websites with relevant advertising. Competitrack’s database includes information about the type of device used to find the ad, jpg files of the ads, ad landing pages, URLs (if available), and general information (eg, site or app where the ad was found, and type [news, weather, shopping, health], date, and brand). We used Competitrack’s database to download general information about prescription drug communications, static images of the full communication, and images of the landing pages (jpg files). Although a few of the mobile communications were interactive, we chose to use a “snapshot in time” approach to capture and code communications and landing pages at 1 point in time to avoid problems of replicability. This method allowed 2 coders to analyze identical ads and landing pages to calculate interrater reliability. We identified 266 mobile communications from May 2012 to May 2014.

We selected eligible mobile communications using the following criteria: (1) the communication and the landing page had to be viewable, (2) only 1 communication per brand could be included, (3) the promoted branded prescription drug had to be regulated by FDA’s Center for Drug Evaluation and Research (CDER) [21], and (4) the communication had to be directed at consumers. We excluded 178 duplicates, plus 22 that were not prescription products regulated by CDER, and 9 due to landing page or mobile screenshot viewing issues. We excluded an additional 6 communications during coding: 4 targeted health care providers rather than consumers, 1 had no mobile communication included, and 1 had no landing page. This resulted in a sample of 51 mobile communications (Table 1).

**Table 1:** Sample characteristics of mobile communications promoting prescription drugs (N=51).

Drug name	Source	Source type	Device	Year
Abilify	CBS News	App	iPhone	2013
Androgel	CNN	Website	iPad	2013
Axiron	Fox News	Website	iPad	2012
Botox	WeatherBug	App	iPhone	2013
Bydureon	AroundMe	App	Android Phone	2014
Celebrex	Fox News	App	iPhone	2013
Chantix	About.com	Website	iPad	2013
Cialis	WeatherBug	Website	Android Phone	2012
Complera	CNBC	Website	Android Phone	2013
Copaxone	eHow	Website	iPhone	2014
Cymbalta	CNN	Website	Android Phone	2013
Dexilant	Dictionary.com	App	Android Phone	2013
Diclegis	TheStreet	App	Android Phone	2013
Dulera	Weatherbug	Website	Android Phone	2012
Eliquis	Fox News	Website	iPad	2013
EpiPen	eHow	Website	iPhone	2013
Evista	Weather.com	Website	iPad	2013
Finacea	About.com	Website	iPad	2014
Gilenya	MSNBC	Website	iPad	2013
Horizant	About.com	Website	iPad	2013
Incivek	About.com	Website	Android Phone	2012
Intermezzo	New York Times	Website	iPad	2013
Intuniv	Learn Spanish Vocabulary Lite	App	Android Phone	2013
Isentress	About.com	Website	iPad	2013
Januvia	WeatherBug	Website	iPad	2013
Latuda	CareerBuilder.com	Website	iPad	2014
Lovaza	Fox News	Website	iPhone	2013
Lunesta	WeatherBug	App	Android Tablet	2013
Mirena	Fox Sports	Website	iPad	2013
Myrbetriq	eHow	Website	Android Phone	2013
Nasonex	AccuWeather	App	iPad	2012
Nexplanon	Craigslist Mobile	App	Android Phone	2013
NuvaRing	WeatherBug	App	iPhone	2012
Nuvigil	Fox Sports	Website	iPad	2013
ParaGard IUD	Dailymotion	Website	Android Phone	2012
PlanB	TweetCaster	App	Android Phone	2012
Staxyn	Fox News	Website	iPad	2013
Stelara	The Weather Channel	App	iPhone	2013
Strattera	Expedia	Website	iPhone	2013
Stribild	About.com	Website	iPad	2013
Suboxone	Fox News	App	iPhone	2013
Symbicort	Weather.com	Website	iPhone	2013
Synthroid	About.com	Website	iPad	2013
Tamiflu	CBS Local YourDay	App	iPhone	2012
Velcade	New York Times	Website	iPhone	2013
Vesicare	eHow	Website	iPhone	2013
Viagra	NFL.com	Website	iPad	2013
Vyvanse	Craigslist Mobile	App	Android Phone	2013
Xarelto	AOL	Website	iPad	2013
Xeljanz	CNN	Website	iPad	2013
Zetonna	AccuWeather	App	iPad	2013

Two raters independently double coded 10% of the sample (5 mobile communications and their related landing pages) to refine the coding scheme, and double coded an additional 10% of the sample to determine interrater reliability (κ=.92 for mobile communication codes and κ=.83 for landing page codes). Disagreements were resolved through discussion. We coded the type of mobile communications and landing pages, the prominence and placement of benefit and risk information, and the presence of links. The type and amount of risk and benefit information in each communication was determined by examining the FDA-approved product labeling for that product. We coded the part(s) of the landing page that were viewable on the screenshot (see Multimedia Appendix 1 for examples). In cases where partial information was visible and ended mid sentence or a capture included additional information that could be accessed through scrolling, as indicated by the presence of a scroll bar, we made the reasonable assumption that it continued below the available screenshot.

## Results

### Mobile Communication Content and Format

Of the 51 mobile communications we coded, 41% (21/51) were product claim communications, 22% (11/51) were reminder communications, and 37% (19/51) were help-seeking communications (see Multimedia Appendix 2 for examples). Scrolling was needed to see all the benefit information on 24% (5/21) of the product claim mobile communications. In contrast, scrolling was needed to see all the risk information on all but 1 of the product claim mobile communications (20/21, 95%). Of the 21 communications, 2 (10%) had additional benefit information available with a link or tab, and 1 (1/21, 5%) had additional risk information available with a link or tab. Of the 19 product claim communications that presented both benefits and risks, 95% (18/19) presented benefits before risks, 47% (9/19) used a bigger font for benefits than for risks, 16% (3/19) used a bigger font size for risks, and 37% (7/19) used the same size font for both.

Of the 51 mobile communications, 37 (72%) included links or tabs to access additional information. The most common link or tab we coded was to the FDA-approved prescribing information [22] (Tables 2 and 3).

### Landing Page Content and Format

The majority of the mobile communications (35/51, 69%) linked to product claim websites, 25% (13/51) linked to landing pages with the drug name and benefit information without visible risk information, and 6% (3/51) linked to landing pages with the drug name and risk information without visible benefit information. Some help-seeking and reminder communications linked to landing pages with the drug name and benefit information without visible risk information (Table 4).

**Table 2:** Types of links and tabs (N=51).

Type of link or tab	Definition	Mobile promotional communication, % (n)	Landing page, % (n)
Prescribing information	A compilation of information about the product, written for the health care practitioner audience, approved by the FDA^a^, based on the agency’s thorough analysis of the new drug application or biologics license application submitted by the applicant. This labeling contains information necessary for safe and effective use.	37 (19)	82 (42)
MedGuide	FDA-approved information that comes with some prescription medicines, determined to pose a serious and significant public health concern. The MedGuide is designed to help patients avoid serious adverse events. MedGuides are a specific category of Patient Labeling.	22 (11)	53 (27)
FDA website	www.fda.gov or www.fda.gov/medwatch	14 (7)	16 (8)
Patient information	Labeled as information for patients, patient information, or patient package insert.	6 (3)	29 (15)

^a^FDA: US Food and Drug Administration.

**Table 3:** Types of links and tabs by type of mobile promotional communication.

Type of link or tab	Type of communication, % (n)		
	Product claim (n=21)	Reminder (n=11)	Help seeking (n=19)
Prescribing information	81 (17)	18 (2)	0 (0)
MedGuide	52 (11)	0 (0)	0 (0)
FDA website	33 (7)	0 (0)	0 (0)
Patient information	14 (3)	0 (0)	0 (0)

**Table 4:** Type of mobile promotional communication by type of landing page (N=51).

Type of communication	Type of landing page, % (n)			Total
	Product claim (name, benefits, and risks)	Drug name and benefits	Drug name and risks	
Product claim	29 (15)	12 (6)	0 (0)	41 (21)
Reminder	14 (7)	6 (3)	2 (1)	22 (11)
Help seeking	25 (13)	8 (4)	4 (2)	37 (19)
Total	69 (35)	25 (13)	6 (3)	100 (51)

Nearly all (48/51, 94%) of the landing pages had visible benefit information, and 88% (45/51) had additional benefit information available with a link or tab. Approximately three-quarters of landing pages had visible risk information (38/51, 74%) and 86% (44/51) had additional risk information available with a link or tab. Scrolling was needed to see all the benefit information on 8% (4/51) of the landing pages. In contrast, scrolling was needed to see all the risk information on 51% (26/51) of the landing pages.

Of the 35 landing pages with both benefit and risk information, 71% (25/35) presented benefits before risks, 51% (18/35) used a bigger font for benefits than for risks, 3% (1/35) used a bigger font size for risks, and 46% (16/35) used the same size font for both.

Nearly all the landing pages (48/51, 94%) included links or tabs to access additional information. The most common link or tab we coded was to the FDA-approved prescribing information (Table 2).

## Discussion

DTC marketing can affect important public health outcomes [23-25], and mobile DTC promotion continues to increase exponentially [26]. Therefore, it is important to understand the content of this promotion. Surprisingly, 22% of mobile communications were reminder communications, a higher proportion than expected given the scarcity of reminders overall. An informal survey of DTC television ads reveals almost no reminder ads in that medium, which may be a direct result of the Pharmaceutical Research and Manufacturers of America DTC guidelines that encourage DTC ads that name a product to also include both risk and indication information [27]. Reminder communications may be suited to the mobile space because they do not have to accommodate benefit and risk information. Enforcement in other space-limited Internet venues likely also affected the types of mobile communications used [28]. Despite FDA guidance in this area [9], sponsors who remain unsure about how to implement a fairly balanced product claim promotion in this medium may opt instead to use a reminder communication to link to the drug’s promotional website [29].

When mobile communications included both benefits and risks, benefits appeared to be more prominent and easily accessible than risks, although we did not test for significant differences. This echoes prior research across a broad range of marketing platforms and raises concerns about whether fair balance requirements are being met in the mobile marketing of prescription drugs in the United States. Similarly, and consistent with past research, benefits also appeared to be more prominent than risks on landing pages [18,20]. It may be that the apparent difference in scrolling needed is because there is often more information in the risk section of approved labeling than in the indication section. Regardless, if risk information is available only through scrolling or a link, consumers may be less likely to access it and may miss important information [30]. Sponsors should ensure that benefit and risk information in mobile communications and on landing pages is equally prominent and accessible to address fair balance.

Some help-seeking and reminder communications linked to a website with the drug name and benefit information but without visible risk information. The FDA has expressed concerns about linking a drug to an indication without accompanying risk information [31]. Moreover, research has shown that consumers confuse disease information with branded prescription drug information when the two are presented together [32] or associated by link [33]. Situations where reminder or help-seeking communications are linked to full product claim websites may require extra care to ensure the information is truthful, balanced, and nonmisleading.

### Limitations

First, the dataset we used consisted mostly of static screenshots. Thus, we could not code interactive features or information that could be viewed only by scrolling. If the screenshot consisted only of the “above the fold” section of a landing page, we could not determine whether risks or benefits were presented “below the fold.” Second, content analysis allows for in-depth analysis of information, but it cannot provide information on how often consumers view mobile communications, what information they understand, or how often they view the landing page. Third, Competitrack did not have an established protocol regarding the orientation of the device. This was done at the discretion of the capture person, but some apps presented the page in landscape by default. As a result, there could potentially be differences in what was captured in a screenshot depending on the device.

### Conclusions

Consumers are increasingly going online for health information, and as digital marketing for prescription drugs is increasing, consumers are more likely to encounter these mobile communications. At the same time, lack of fair balance generally, and minimization of risk information in particular, continue to be frequently cited in FDA letters [34]. Our findings suggest that sponsors continue to struggle with these issues across promotional platforms. Recent FDA guidance on character space-limited platforms [8,9] may help sponsors navigate these issues. The results of this analysis, particularly with respect to the persistent lack of fair balance, may provide useful information to regulators as they consider the available data applicable to policy. Additionally, continued surveillance of DTC marketing online and in mobile communications is needed to ensure that consumers have access to fairly balanced, accurate, and nonmisleading information about medical products.

## Appendix

Multimedia Appendix 1 Landing page examples.

Multimedia Appendix 2 Type of mobile promotional communication in sample.

## Abbreviations

CDER: Center for Drug Evaluation and Research
DTC: direct-to-consumer
FDA: US Food and Drug Administration
