# Supplementary material for: Direct-to-Consumer Promotion of Prescription Drugs on Mobile Devices: Content Analysis
Source: J Med Internet Res. 2017 Jul 4;19(7):e225. doi: 10.2196/jmir.7306 (PMC5516101; doi:10.2196/jmir.7306)
Supplement: Multimedia Appendix 1 [file jmir_v19i7e225_app1.pdf]

Landing page examples

<table>
<tr><th>Type of information visible</th><th>Example</th></tr>
<tr><td>Drug name, benefits, and risks</td><td>
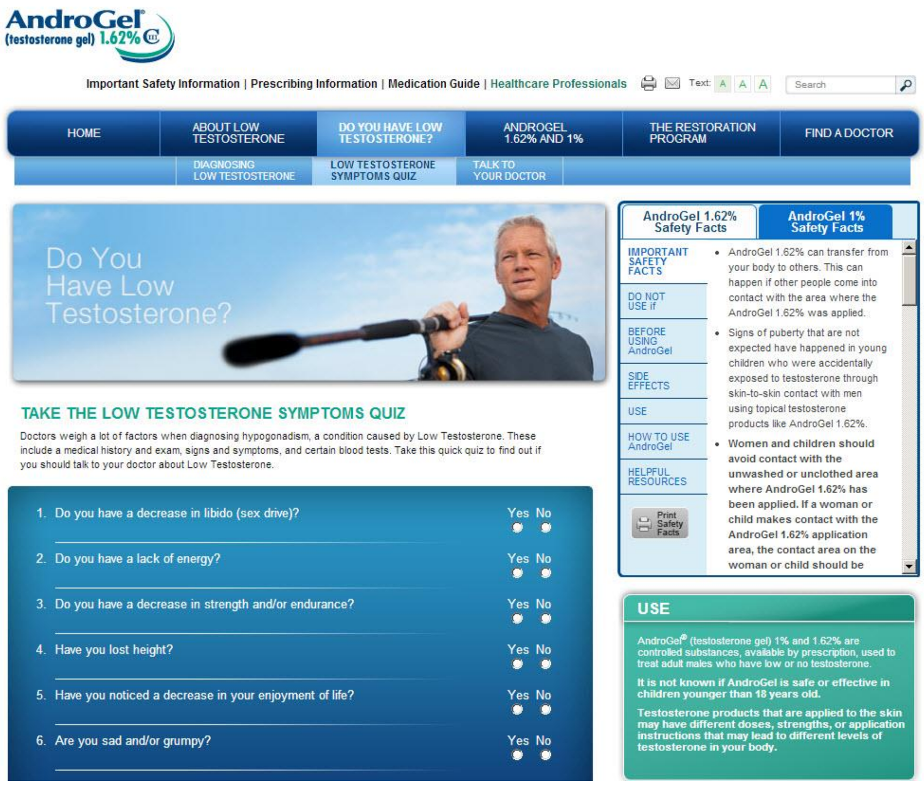

</td></tr>
</table>

Drug name and benefits	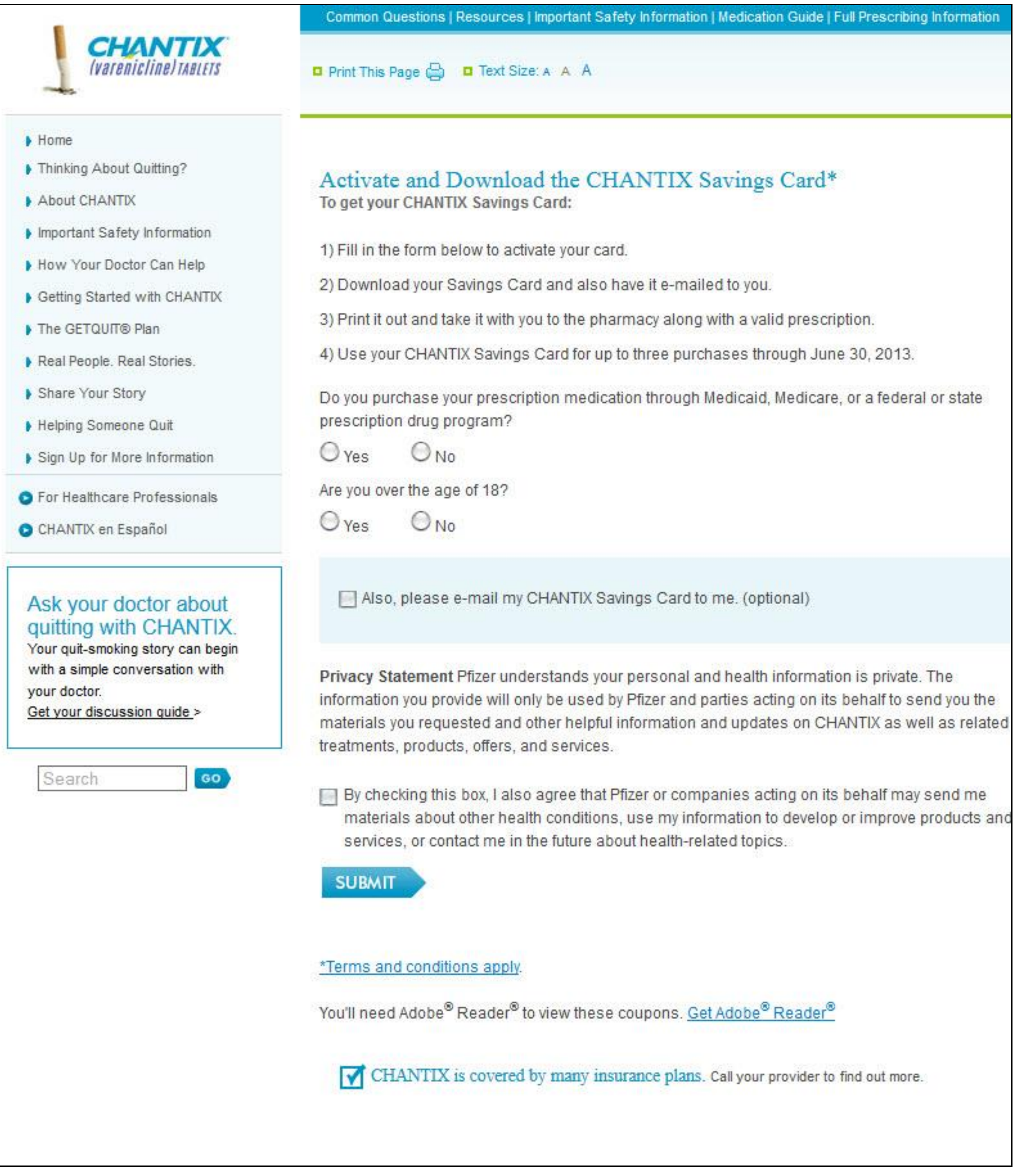

<table>
<tr>
<td>Drug name and risks</td>
<td>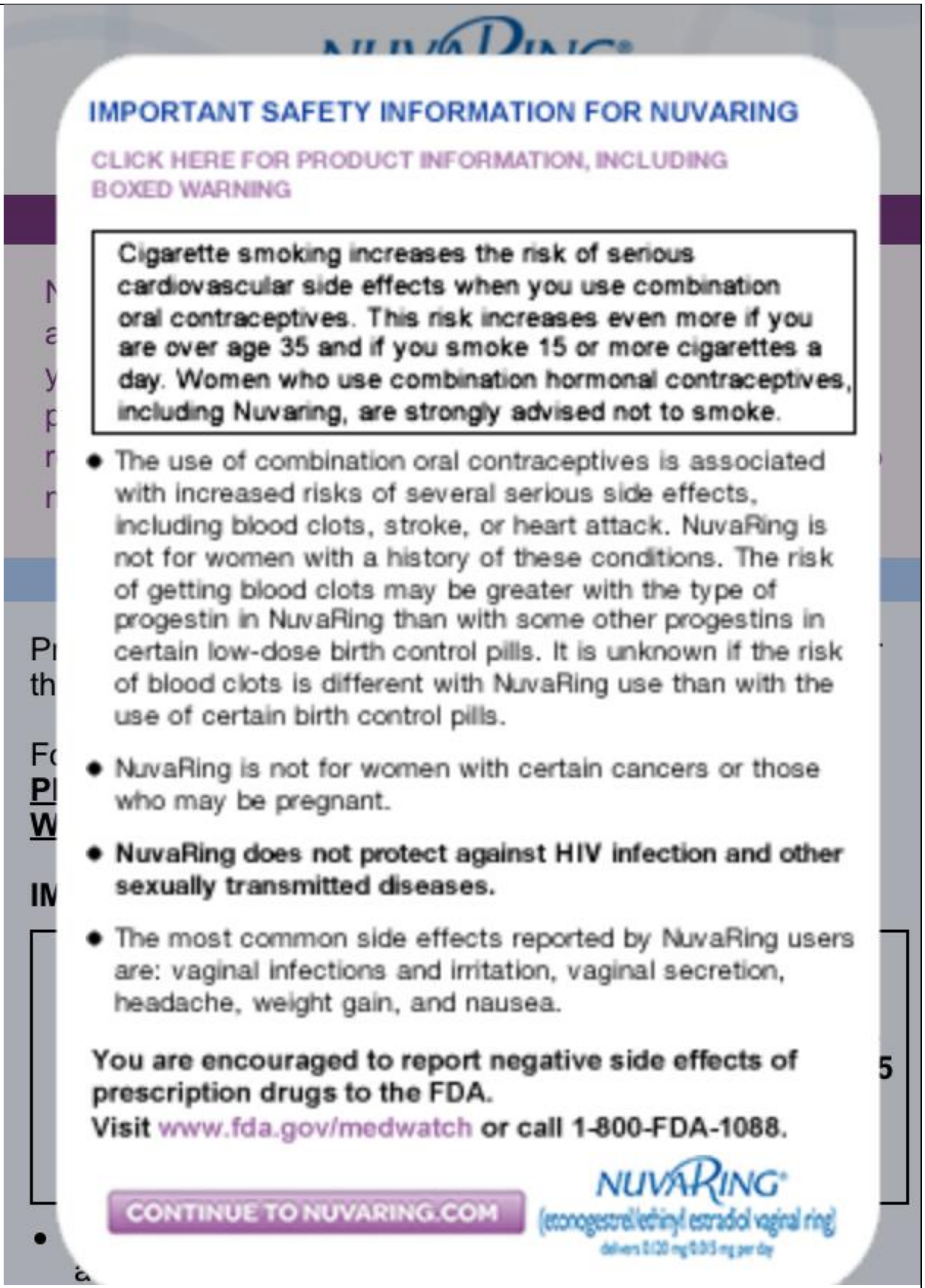
</td>
</tr>
</table>

Note: Note: Use of brand names does not imply endorsement by FDA. Images from Competitrack, https://markettrack.com/advertising.
