# Supplementary material for: Direct-to-Consumer Promotion of Prescription Drugs on Mobile Devices: Content Analysis
Source: J Med Internet Res. 2017 Jul 4;19(7):e225. doi: 10.2196/jmir.7306 (PMC5516101; doi:10.2196/jmir.7306)
Supplement: Multimedia Appendix 2 [file jmir_v19i7e225_app2.pdf]

Type of mobile promotional communication in sample

Type of communication	Definition	Example
Product claim	Includes the drug name, drug benefits, drug risks	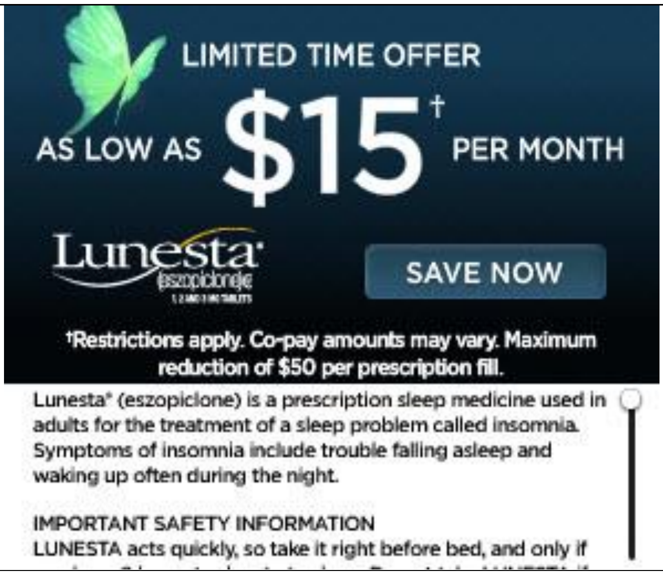
Reminder	Includes only the drug name	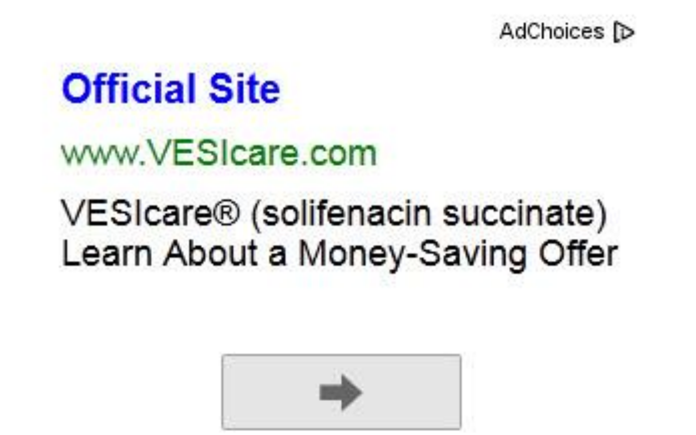
Help seeking	Presents information about the medical condition the drug treats and does not include the drug name	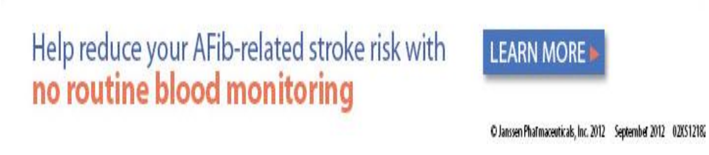

Note: Use of brand names does not imply endorsement by FDA. Images from Competitrack, https://markettrack.com/advertising.
